# Cryo-EM structure-based discovery of etravirine as a specific inhibitor of PRMT5/pICln protein-protein interaction for prostate cancer treatment

**DOI:** 10.1080/14756366.2026.2727844

**Published:** 2026-09-08

**Authors:** Zhixia Chi, Xueyong Xu, Zhihang Shen, Xuehong Deng, Bennett D. Elzey, Chenglong Li, Wen Jiang, Chang-Deng Hu

**Affiliations:** a Department of Chemistry, Purdue University, West Lafayette, Indiana, USA; b Purdue University Interdisciplinary Life Sciences Graduate Program, Purdue University, West Lafayette, Indiana, USA; c Department of Biological Sciences, Purdue University, West Lafayette, Indiana, USA; d Department of Medicinal Chemistry, College of Pharmacy, University of Florida, Gainesville, FL, USA; e Department of Medicinal Chemistry and Molecular Pharmacology, Purdue University, West Lafayette, Indiana, USA; f Department of Comparative Pathobiology, Purdue University, West Lafayette, Indiana, USA; g Purdue Institute for Cancer Research, Purdue University, West Lafayette, Indiana, USA; h Department of Biochemistry and Molecular Biology, Pennsylvania State University, University Park, Pennsylvania, USA

**Keywords:** PRMT5, pICln, prostate cancer, protein-protein interaction inhibitor, cryo-EM

## Abstract

Protein arginine methyltransferase 5 (PRMT5) is overexpressed in many cancers and correlates with poor patient survival. In prostate cancer, PRMT5 cooperates with its cofactor pICln to promote tumour growth by epigenetically activating androgen receptor (AR) expression. Using a near-atomic cryo-EM structure of PRMT5/MEP50/pICln complex, we identified a previously undefined, pICln-specific protein-protein interaction (PPI) interface on PRMT5, termed P4I. Structure-based virtual screening identified the FDA-approved compound etravirine as a binder to this site. BiFC, Co-IP, and PLA assays confirmed that etravirine disrupts PRMT5/pICln interaction. A cryo-EM structure of PRMT5/MEP50/etravirine further validated on-target binding at P4I. Functionally, etravirine reduced prostate cancer cell proliferation, inhibited tumour growth, and downregulated AR and AR-V7 expression in cells and in mouse models. These results demonstrate that the unique P4I interface is a promising therapeutic target and that etravirine serves as a proof-of-concept lead compound for exploring the potential of P4I-targeted strategies in prostate cancer.

## Introduction

Protein arginine methyltransferase 5 (PRMT5) is a class II protein arginine methyltransferase (PRMT)^1^. Aberrant overexpression of PRMT5 is observed in many malignancies and is accompanied by poor clinical outcomes^2^. PRMT5 catalyses symmetric dimethylation of arginine residues of histone substrates. As such, it can regulate the transcription of genes by dimethylation of H4R3, H3R2, H3R8, and H2AR3 histones^3^. Increasing numbers of non-histone substrates of PRMT5 have also been identified, and they are involved in a variety of cellular processes, such as ribosomal biogenesis, pre-mRNA splicing, DNA damage response, immune response, and cell signalling that could determine cell fate^4^. In recent years, multiple strategies of PRMT5 inhibition have been explored to develop therapeutic compounds, with several having advanced to clinical trials^5^. Most of these inhibitors target the active site in the C-terminal catalytic domain of PRMT5, either the methyl donor S-adenosyl methionine (SAM) binding pocket, the protein/peptide substrate binding pocket, or both pockets simultaneously, thereby abrogating PRMT5 function^6^. However, since PRMT5 is critical for normal cell homeostasis and survival, targeting these active sites may cause severe side effects and limit the therapeutic window for cancer treatment. Consequently, there is a need for the development of small molecules capable of inhibiting specific aspects of PRMT5 functions.

The substrate-binding specificity and methyltransferase activity of PRMT5 are regulated through multiple mechanisms, including interactions with co-factor proteins like MEP50, COPR5, pICln, and RioK1. For instance, MEP50 is critical for PRMT5-catalyzed histone methylation while COPR5 modulates the substrate specificity of Histone H4; pICln regulates spliceosome assembly, and RioK1 competes with pICln to recruit nucleolin^7–10^. Recently, innovative strategies to regulate the function of PRMT5 in cancer cells have been reported. Sellers and Waldmann groups independently identified a highly conserved linear peptide sequence, PRMT5 binding motif (PBM), bound to a shallow groove of the PRMT5 TIM-barrel domain^11^^,^^12^. The PBM is a shared interaction site for pICln, RioK1, and COPR5. Mutation of the groove impaired the viability of MTAP-null cancer cell lines, strongly suggesting that targeting PPIs between PRMT5 and its protein co-factors presents a promising therapeutic approach to treat various types of cancers^11^.

Prostate cancer (PC) is the most common cancer and the second leading cause of cancer-related death in men^13^. Androgen receptor (AR) is expressed in nearly all primary prostate cancers and is critical for prostate cancer development and progression^14^. Androgen deprivation and functional inhibitors of AR are utilised to treat PC. However, prostate tumour cells evolve resistance to these treatments by expressing a constitutively active, ligand-independent variant of AR known as AR-V7. We have previously reported that PRMT5 regulates AR expression and promotes cell growth of castration-resistant prostate cancer (CRPC), both *in vitro* and *in vivo*^15^^,^^16^. Subsequently, we also determined that pICln cooperates with PRMT5 to promote AR transcription in CRPC via dimethylation of H4R3 at the proximal AR promoter^16^. Taken together, these results suggest targeting PRMT5/pICln PPI could be a potential therapeutic approach for prostate cancer treatment.

In this work, we started by determining a near-atomic cryo-EM structure of the PRMT5/MEP50/pICln complex. Our structure revealed a previously undefined PPI site unique to the PRMT5/pICln interface, distinct from the PBM. A structure-based virtual screening identified etravirine, a compound originally developed for the treatment of HIV^17^, as a non-covalent inhibitor targeting the unique PPI site of PRMT5/pICln. Our bimolecular fluorescence complementation (BiFC), Co-immunoprecipitation (Co-IP), and proximity ligation assay (PLA) results validated that etravirine effectively disrupted the PRMT5/pICln interaction in prostate cancer cells. Additionally, we demonstrated that etravirine inhibited AR, and AR-V7 expression, as well as prostate cancer cell proliferation in cell and mouse models. Overall, our structural and functional studies indicate that etravirine is a promising lead compound with the potential to be a first-in-class prostate cancer treatment by specifically inhibiting PRMT5/pICln PPI discovered in this work.

## Results

### A unique PRMT5/pICln PPI interface revealed by cryo-EM

**Figure 1.**
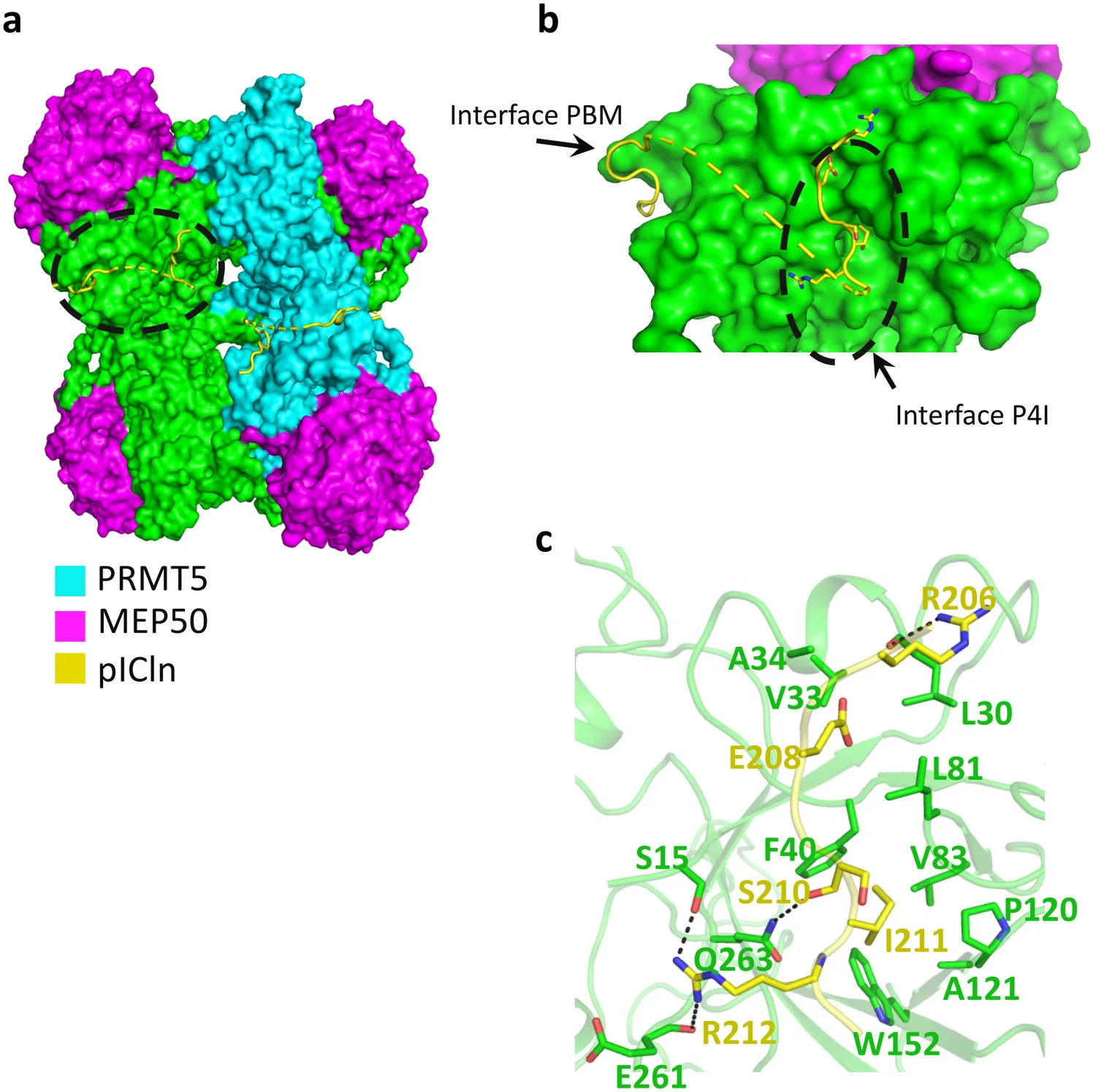
A new PRMT5/pICln interaction interface, P4I, identified in our Cryo-EM structure of PRMT5/MEP50/pICln complex. **a,** Overall structure of PRMT5/MEP50/pICln complex. **b,** Close-up view of the interfaces between PRMT5 and pICln. Interface PBM was previously identified between PBM and the shallow groove, whereas interface P4I is the newly identified interface in our cryo-EM structure. **c,** Close-up view of the molecular interaction between PRMT5 and pICln at the P4I interface.

To elucidate the interaction between PRMT5 and pICln, we reconstituted the PRMT5/MEP50/pICln ternary complex with full-length, co-expressed proteins and determined the near-atomic cryo-EM structures (Figure 1a, Supplementary Figure 1 and 2). There are two types of structures with similar abundance in our data, with different PRMT5/MEP50 copy numbers. The first type corresponds to the (PRMT5)_4_(MEP50)_4_(pICln)_4_ complex at 3.03 Å resolution, in which the stoichiometry of (PRMT5)_4_(MEP50)_4_ has been reported in the crystal and cryo-EM structures of PRMT5/MEP50 complex^18–20^. Unexpectedly, cryo-EM analysis also revealed a second complex, (PRMT5)_4_(MEP50)_3_(pICln)_4_ at 3.59 Å resolution (Supplementary Figure 2; Table S1). In the (PRMT5)_4_(MEP50)_4_(pICln)_4_ structure, the four protomers of the hetero-dodecamer form D2 symmetry, with each protomer consisting of one molecule each of PRMT5, MEP50, and pICln. The structure of the (PRMT5)_4_(MEP50)_3_(pICln)_4_ complex is nearly identical to that of (PRMT5)_4_(MEP50)_4_(pICln)_4,_ with the absence of one MEP50 subunit as the primary structural difference. The structural analysis and discussion below thus focus on the (PRMT5)_4_(MEP50)_4_(pICln)_4_ structure_._ Unambiguous models could be built for almost the entire polypeptides of PRMT5 and MEP50, except for a few flexible loops. The overall PRMT5/MEP50 structure and each protomer structure were in close agreement with counterparts of the previously published crystal and cryo-EM structures of PRMT5/MEP50, except for a slightly more open orientation between the TIM barrel and catalytic domains of PRMT5 in our structure (Supplementary Figure 3).

However, although we reconstituted the complex using full-length pICln containing 237 residues, only the densities for residues 205–213 and residues 224–234 are well resolved, suggesting that other regions of pICln may not directly interact with PRMT5/MEP50 or could exhibit dynamic positioning relative to the well-resolved PRMT5/MEP50 and the C-terminal region of pICln. In our structure, pICln residues 224–234 occupy the shallow groove of the PRMT5 TIM-barrel domain that was previously identified as the binding site for the conserved PBM shared among PRMT5 co-factors, including pICln, RioK1, and COPR5. The pICln residues 205–213 in our structure were found to bind PRMT5 at an adjacent interface (Figure 1b), which we will refer to as P4I (i.e. PRMT5/pICln PPI). At this previously undefined interface, the residues 205–213 of the pICln short loop form a network of van der Waals and hydrogen bonding interactions with PRMT5 (Figure 1c). Glu208 inserts into a pocket of PRMT5 composed of Leu30, Val33, Ala34 and Leu81, and forms van der Waals interactions with these surrounding residues. The hydrophobic side chain of Ile211 of pICln snugly fits into a deep hydrophobic pocket of PRMT5, the floor of which is composed of side chains of Phe40, Val83, Pro120, Ala121, and Trp152 of PRMT5. This extensive complementary hydrophobic interface is orchestrated by multiple hydrogen bonds between the pICln and PRMT5 residues: Arg212:NH1 of pICln and Ser15:OG of PRMT5, Arg212:NH2 of pICln and the main-chain carbonyl group of Glu261 of PRMT5, Arg206:NH2 of pICln and the main-chain carbonyl groups of Leu30 of PRMT5, and Ser210 main-chain amino group of pICln and Gln263:NE2 of PRMT5.

Although the PBM of pICln has been shown to be necessary and sufficient for its binding to PRMT5, our structural data highlight the significance of pICln residues 205–213, especially Ile211 and Arg212, in stabilising the interaction with PRMT5. This suggests that our newly discovered interface P4I may be as essential, if not more so, for the pICln and PRMT5 interaction. While the PBM is shared among pICln, COPR5, and RioK1, sequence alignment revealed no similarity between residues 205–213 of pICln and those of COPR5 or RioK1, indicating that the P4I site is unique to the PRMT5/pICln interaction. Furthermore, pICln residues 205–213 are absent in invertebrates, whereas residues 205–208 are conserved across vertebrates. Notably, the full P4I segment (residues 205–213) is exclusively conserved among Reptilia and higher vertebrates, indicating that this interaction motif evolved during the emergence of higher vertebrates (Supplementary Figure 4).

### Identification of etravirine as a binder at the P4I site by structure-based virtual screening

We have demonstrated that our newly identified P4I interface is unique to the PRMT5/pICln PPI, and the deep hydrophobic pocket region of PRMT5 is critical for the PRMT5/pICln interaction. To identify inhibitors targeting this interface, specifically the deep hydrophobic pocket, to disrupt PRMT5/pICln PPI without affecting PRMT5/COPR5 or PRMT5/RioK1 PPIs, we conducted a virtual screening targeting the P4I pocket on the PRMT5 N-terminal TIM-barrel domain from the (PRMT5)_4_(MEP50)_4_(pICln)_4_ complex at 3.03 Å resolution. Our in-house small molecule compounds library, comprising selected compounds from DrugBank, Lopac1280, ChemDiv150K, was screened using Glide SP. After visually inspecting the top 50 compounds with the best affinity scores for their interactions with PRMT5, focusing on the most optimal hydrophobic/aromatic/hydrogen bonding interactions, five compounds were selected for experimental verification. These five compounds were 5-[(4-Chlorophenyl)sulfanyl]-1,2,3-thiadiazole-4-carboxylic acid, AG205, N-acetyl-β-D-glucosamine-thiazoline, etravirine, and Turofexorate isopropyl. Among them, bimolecular fluorescence complementation (BiFC) results show that etravirine had the best potency to inhibit PRMT5/pICln interactions and was thus chosen for further characterisations. Etravirine (CAS number: 269055–15-4; IUPAC chemical name: 4-[[6-amino-5-bromo-2-[(4-cyanophenyl)amino]-4-pyrimidinyl]oxy]-3,5-dimethylbenzonitrile) is a non-nucleoside reverse transcriptase inhibitor (NNRTI) approved by the FDA in combination with other antiretrovirals for treating HIV-1 infection in treatment-experienced adults and paediatric patients aged 2 years and older with strains resistant to (protease inhibitors) PIs, nucleoside reverse transcriptase inhibitors (NRTIs), and other NNRTIs. The posology of etravirine is 200 mg administered twice daily^21^.

### Confirmation of PRMT5/etravirine binding by surface plasmon resonance (SPR)

**Figure 2.**
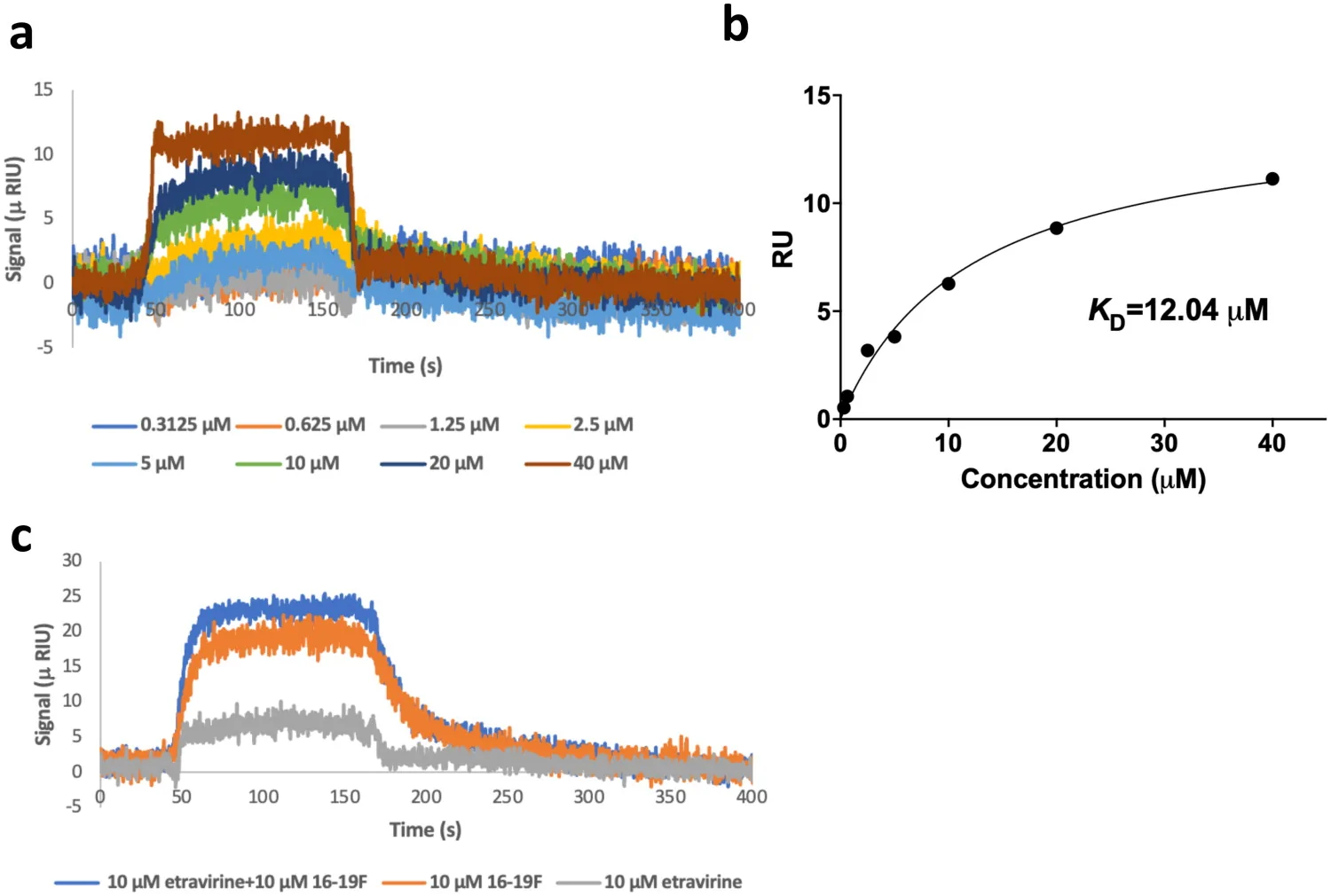
Etravirine binds to the PRMT5/MEP50 complex at a site distinct from the substrate pocket. **a,** SPR sensorgram of etravirine binding to PRMT5/MEP50 protein complex. **b,** Concentration-dependent binding of etravirine to PRMT5/MEP50 based on SPR measurements from (**a**). **c,** etravirine and 16–19 F binding to PRMT5/MEP50 show additive instead of competitive binding, indicating that etravirine binds at a site distinct from the substrate binding site of 16–19 F.

To verify the computationally predicted binding of etravirine to PRMT5, we performed surface plasmon resonance (SPR) analysis. SPR analysis revealed a clear binding interaction between etravirine and the PRMT5/MEP50 complex, with a binding affinity (i.e. dissociation constant, Kd) of 12.0 µM (Figure 2a, b). To confirm that etravirine does not bind to the PRMT5 active site, competitive SPR binding assays were conducted. A mixture of 10 µM etravirine and a saturating concentration of 16–19 F (an in-house synthesised MTA-cooperative inhibitor)^22^ produced an additive signal, indicating that etravirine and 16–19 F bind to distinct binding sites on PRMT5 (Figure 2c). This study highlights etravirine as a promising inhibitor targeting the unique PRMT5/pICln PPI interface P4I, with potential therapeutic implications for selectively disrupting this interaction.

### Etravirine specifically inhibits PRMT5/pICln interaction in prostate cancer cells

To confirm that the newly identified P4I site revealed by our cryo-EM structure is critical for PRMT5/pICln interaction, we employed the BiFC assay to validate PRMT5/pICln interaction in prostate cancer cells. BiFC is a well-established method that has been extensively utilised to visualise as well as to screen PPIs in live cells and animals^23^, and the prostate cancer cell lines were extensively utilised by us to explore the biological roles of PRMT5 and pICln^15^^,^^16^^,^^24^^,^^25^. In the BiFC assay, the N-terminal fragment of Venus fluorescent protein (VN, residues 1–154) was fused to the N-terminus of PRMT5, while the C-terminal fragment of Venus fluorescent protein (VC, residues 155–238) was fused to the N-terminus of pICln. Next, we utilised BiFC to determine whether etravirine could disrupt PRMT5/pICln interaction in prostate cancer LNCaP cells in the absence or presence of etravirine. We observed that etravirine reduced the yellow fluorescent signal arising from the complemented Venus fluorescent protein due to PRMT5/pICln PPI, with the cyan fluorescent signal of Cerulean fluorescent protein (CFP) as a reference control (Supplementary Figure 5a). Quantitative analysis of BiFC images indicated that etravirine significantly inhibited PRMT5/pICln interaction (Supplementary Figure 5b). These findings suggest that etravirine efficiently disrupts PRMT5/pICln PPI in prostate cancer cells, consistent with our SPR results. These BiFC results support the notion that the newly defined P4I interface is unique to and critical for PRMT5/pICln interaction.

**Figure 3.**
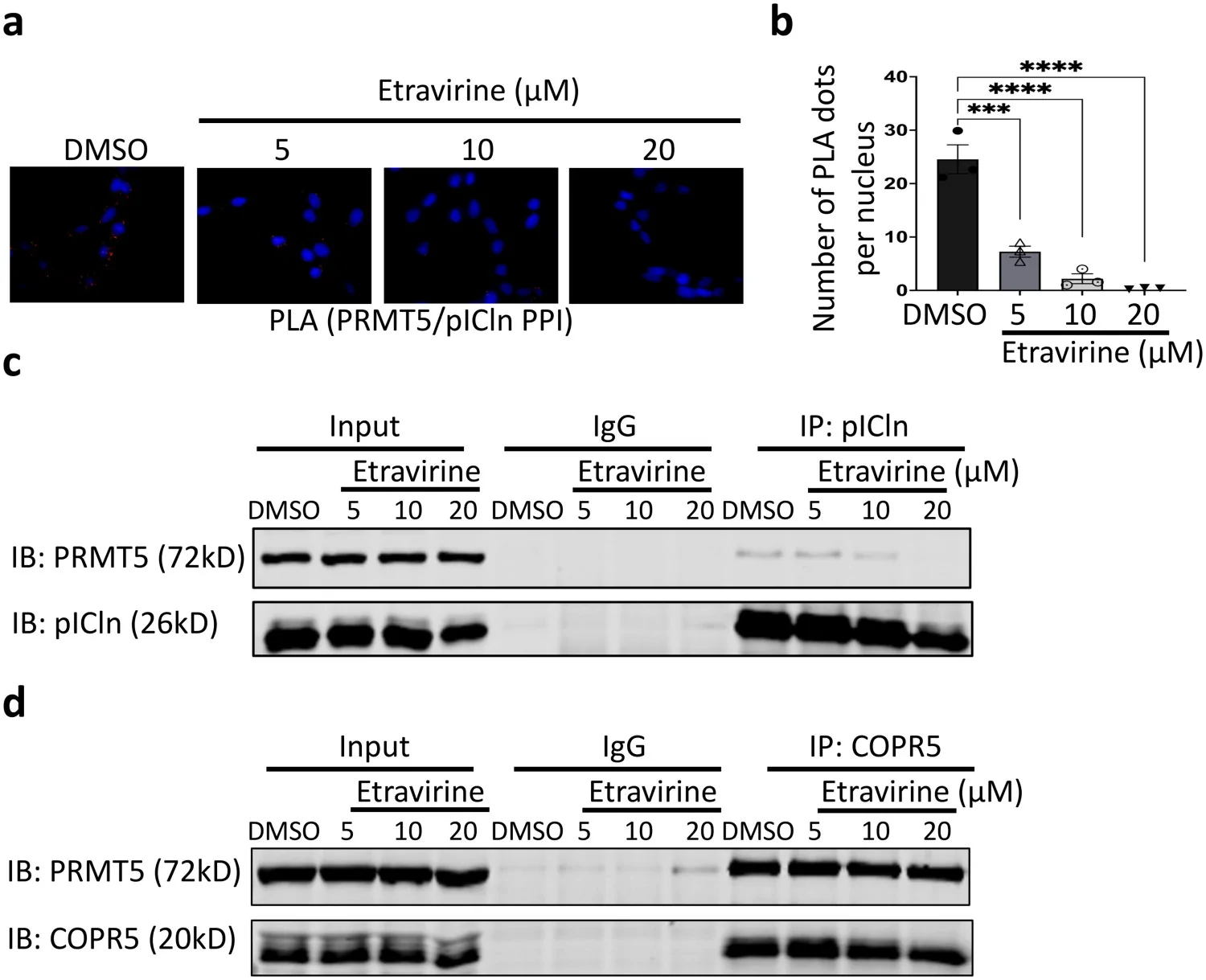
Etravirine selectively inhibits PRMT5/pICln interaction in prostate cancer cells. **a,** Representative PLA (red) images in LNCaP cells acquired at 40× magnification, treated with different concentrations of etravirine or DMSO. **b,** Quantification of the effect of etravirine on PRMT5/pICln interaction from **(a)**. **c,** Representative Western blot images of Co-IP of pICln and PRMT5 in 22Rv1 cell lysates after treatment with either DMSO or etravirine at the indicated concentrations (5, 10, and 20 μM). **d,** Representative Western blot images of Co-IP of COPR5 and PRMT5 in 22Rv1 cell lysates with the same treatments in **(c)**. All the above images are representative of three independent experiments. For PLA quantification, results are mean ± SEM from three independent experiments. Ordinary one-way ANOVA was performed to determine statistical significance, ***, *P* < 0.001; ****, *P* < 0.0001.

To investigate the location of endogenous PRMT5/pICln interaction in prostate cancer cells, we utilised proximity ligation assays (PLA) to assess whether etravirine could impede the endogenous PRMT5/pICln interaction. We observed that their interactions predominantly occurred in the cytoplasm of DMSO-treated LNCaP cells, consistent with cytoplasm as the primary expression compartment of PRMT5 and pICln. Furthermore, compared to DMSO control, etravirine treatment led to a dose-dependent reduction of PLA signal in LNCaP cells (Figure 3a, 3b).

To further evaluate whether etravirine disrupts the endogenous PRMT5/pICln complex in prostate cancer cells, Co-IP of endogenous PRMT5 and pICln was performed with or without etravirine treatment in 22Rv1 cells, which is a human prostate carcinoma cell line widely used to study CRPC. In the presence of etravirine, less PRMT5 was co-immunoprecipitated with pICln, compared to DMSO control (Figure 3c). These Co-IP results confirm that etravirine impairs the PRMT5/pICln interaction.

Since etravirine was computationally screened for binding to our newly discovered P4I site unique to PRMT5/pICln interactions, distinct from the previously reported PBM shared by pICln, RioK1 and COPR5, we reasoned that etravirine should specifically perturb PRMT5/pICln interactions in prostate cancer cells, without affecting PRMT5 interaction with other cofactor proteins. To test this, we performed Co-IP of endogenous PRMT5 and COPR5 with or without etravirine treatment in 22Rv1 cells. The Co-IP results demonstrated that etravirine did not affect PRMT5/COPR5 interaction (Figure 3d).

In summary, the results of multiple biophysical and biochemical assays, including SPR, BiFC, PLA, and Co-IP, have collectively demonstrated that etravirine disrupts the unique PRMT5/pICln interaction.

### Cryo-EM confirms etravirine binding at the unique PRMT5/pICln PPI site P4I

To elucidate the structural basis of the interactions between PRMT5 and etravirine, we determined a cryo-EM structure of the PRMT5/MEP50/etravirine complex at an overall resolution of 3.12 Å (Supplementary Figure 6; Table S1). Surprisingly, this structure exhibits a stoichiometry of (PRMT5)_4_(MEP50)_3_, where only one MEP50 displays strong density, while the other two MEP50 show much weaker densities. This is reminiscent of the structure of (PRMT5)_4_(MEP50)_3_(pICln)_4,_ in which there was also a sub-stoichiometric number of MEP50, although all three MEP50 subunits have more even, strong densities. The sub-stoichiometric binding of MEP50 to PRMT5 breaks the D2 symmetry, prompting us to solve the PRMT5/MEP50/etravirine complex structure without imposing any symmetry. Despite the partial loss of MEP50, the map reveals clear densities for most residues of PRMT5 and the remaining MEP50, with no noticeable conformational changes observed in PRMT5 or the bound MEP50.

**Figure 4.**
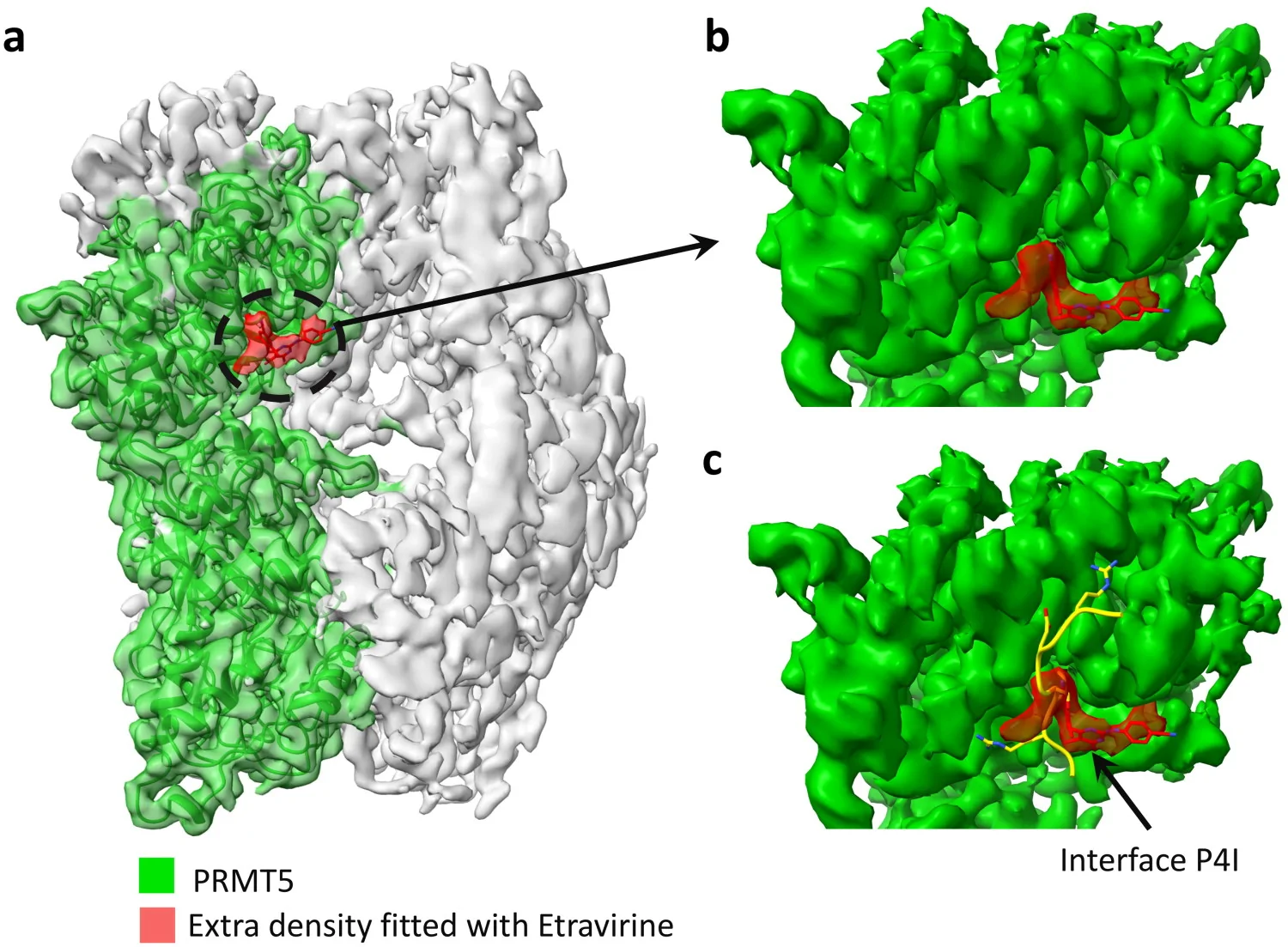
Cryo-EM structure of PRMT5/MEP50/etravirine complex confirms that P4I is the etravirine binding site. **a,** Cryo-EM map of the focused reconstruction of PRMT5/MEP50/etravirine complex around the P4I site. **b,** Close-up view of the PRMT5 and etravirine binding site. **c,** Superimposition of PRMT5/pICln in the structure of PRMT5/MEP50/pICln complex on the cryo-EM map of PRMT5/MEP50/etravirine complex. Etravirine (red) occupies the same P4I binding site of pICln (gold).

Extra densities were readily observed at all four D2-symmetry-related locations of the unique PRMT5/pICln PPI site P4I (Supplementary Figure 6). Since we used the same buffer (10 mM Hepes, pH7.5, and 150 mM NaCl) as in previous crystal and cryo-EM structures of human PRMT5/MEP50 complex, which lacked such densities, we assigned these densities as the bound etravirine. To improve the extra densities to allow more accurate fitting of etravirine, we attempted local 3D classification and local reconstruction after D2 symmetry expansion, which improved the map from 3.12 to 2.93 Å resolution. However, the extra densities were still not sufficiently resolved to unambiguously fit etravirine, likely due to the relatively weak interaction between etravirine and PRMT5 with a binding affinity of 12 µM (Figure 4a). Nevertheless, it is evident that etravirine occupies the same PRMT5 P4I site with a deep hydrophobic pocket, which is critical for PRMT5/pICln interaction (Figure 4b, 4c). Thus, our cryo-EM structure of the PRMT5/MEP50/etravirine complex confirms the binding of etravirine to PRMT5 predicted by virtual screening and suggests that etravirine functions as a competitive ligand for PRMT5, inhibiting the binding of pICln to PRMT5.

### Etravirine suppresses the proliferation of prostate cancer cells

**Figure 5.**
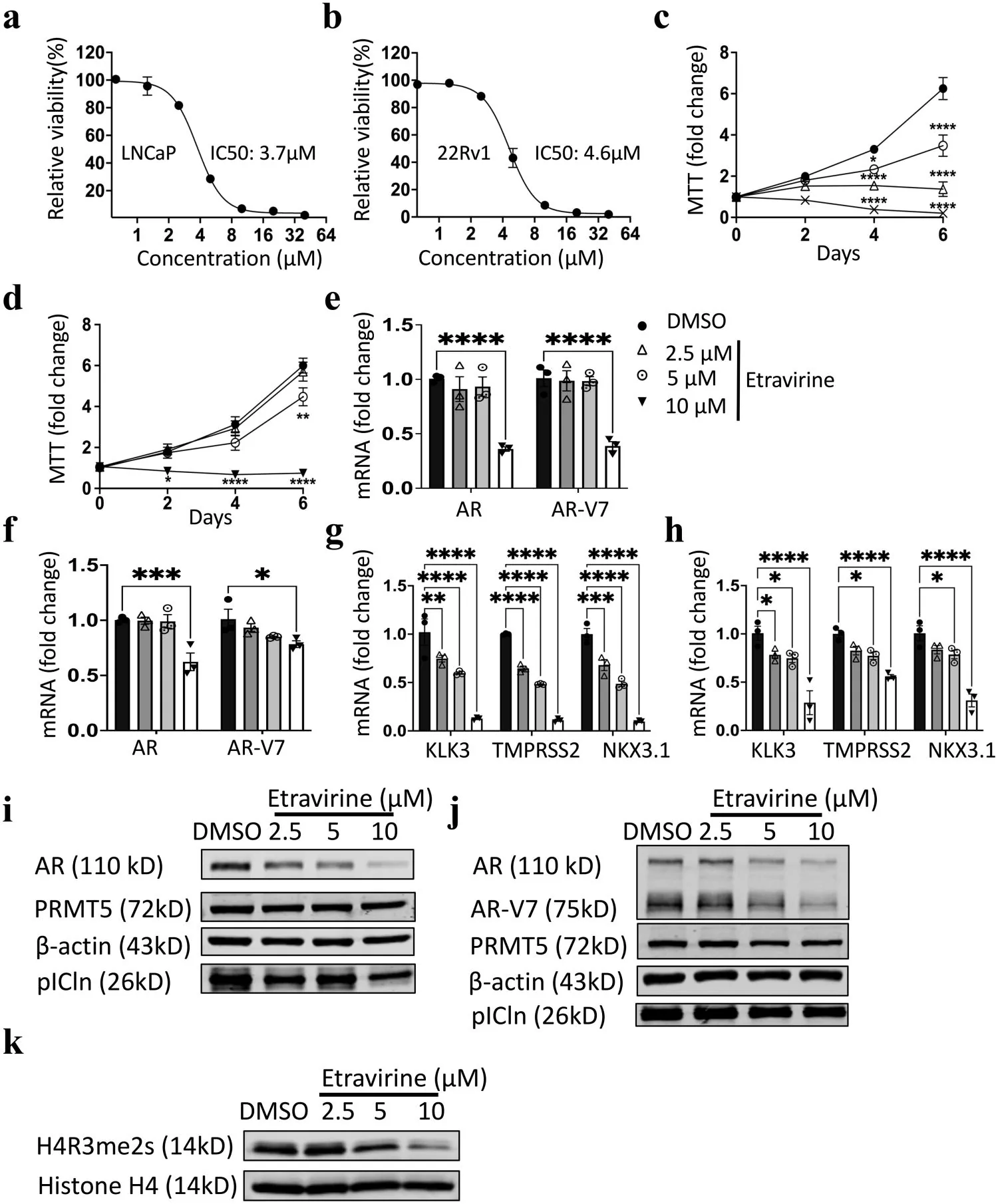
Etravirine inhibits prostate cancer cell proliferation and decreases AR expression. **a**, **b**, Etravirine dose-dependent viability of LNCaP (**a**) and 22Rv1 (**b**) cells. **c, d**, Growth curves (MTT assay) of LNCaP and 22Rv1 cells incubated with etravirine (2.5 μM, 5 μM, 10 μM) or DMSO for 6 days. **e, f**, qPCR quantification of AR gene expression in LNCaP (**e**) and 22Rv1 cells (**f**) after etravirine treatment for 24 h. The four bars in each of the plots in e-f correspond to DMSO, etravirine at 2.5, 5, and 10 μM, respectively. **g, h**, qPCR quantification of AR target genes KLK3, TMPRSS2, and NKX3.1 mRNA expression in LNCaP (**g**) and 22Rv1 cells (**h**). **i, j** Representative Western blot images for LNCaP (**i**) and 22Rv1 cells (**j**) after treatment with DMSO or etravirine for 24 h. **k,** Representative Western blots of H4R3 dimethylation in 22Rv1 cells with or without etravirine treatment. All images are representative of three independent experiments. For MTT and qPCR quantification, results are mean ± SEM from three independent experiments. Two-way ANOVA was performed to determine statistical significance, ns, nonsignificant, *P* > 0.05; *, *P* < 0.05; **, *P* < 0.01; ***, *P* < 0.001; ****, *P* < 0.0001.

We and others have previously reported that PRMT5 expression is essential for prostate cancer cell growth^15^^,^^16^^,^^26^^,^^27^. Recently, we demonstrated that targeting either PRMT5 or pICln inhibited the growth of CRPC cells^16^. Given that etravirine disrupts the PRMT5/pICln interaction, we evaluated the biological effects of etravirine on prostate cancer cell lines, LNCaP and 22Rv1. The cells were treated with etravirine at concentrations ranging from 40 μM to 0.625 μM, or with DMSO as a control. Etravirine inhibited the growth of LNCaP (Figure 5a) and 22Rv1 cells in a dose-dependent manner (Figure 5b) with IC50 values for LNCaP and 22Rv1 at 3.7 μM and 4.6 μM, respectively. Furthermore, the proliferation of LNCaP and 22Rv1 cells exhibited a dose- and time-dependent response to etravirine treatment (Figure 5c, 5d).

### Etravirine suppresses AR and AR-V7 expression in prostate cancer cells

AR is a steroid and nuclear receptor that regulates androgen-related gene expression. It is critical for normal tissue function and the progression of hormone-dependent cancer, such as prostate and breast cancer^27^. It has been reported that PRMT5 cooperates with pICln, rather than MEP50, to promote the growth of AR-positive CRPC cells through epigenetic activation of both AR-FL and AR-V7 transcription^4^. To determine whether etravirine-mediated suppression of cell growth is AR-dependent, we examined AR and AR-V7 mRNA expression in LNCaP cells and 22Rv1 cells. As expected, both cell lines expressed AR and AR-V7 mRNA, with 22Rv1 cells exhibiting significantly higher levels of AR-V7 mRNA compared to LNCaP cells (Figure 5e, 5f), consistent with previous reports^28^. Etravirine significantly reduced AR and AR-V7 transcription in a concentration- and time-dependent manner in both LNCaP and 22Rv1 cells (Figure 5e, 5f), while not affecting PRMT5 and pICln transcription (Supplementary Figure 7a, 7b). To further validate the downregulation of AR mRNA by etravirine, we assessed the expression of AR target genes, including KLK3 (or PSA), TMPRSS2, and NKX3.1, which are regulated by AR and play roles in prostate cancer progression^29^. Etravirine treatment led to a dose- and time-dependent reduction in the mRNA expression of these genes (Figure 5g, 5h). Western blot analysis confirmed that etravirine reduced AR protein expression in LNCaP cells, and decreased both AR and AR-V7 protein expression in 22Rv1 cells, while PRMT5 or pICln expression remained unaffected (Figure 5i, 5j). These findings indicate that the anti-cell growth efficacy of etravirine is AR- and AR-V7-dependent in prostate cancer cells.

Our previous finding suggests that PRMT5 recruits pICln to the AR promoter, and PRMT5/pICln interaction is required for PRMT5-mediated H4R3 dimethylation at the AR promoter to activate AR transcription^16^. To evaluate whether etravirine affects H4R3 dimethylation, we assessed the H4R3me2s expression in 22Rv1 cells with or without etravirine treatment. Western blot results showed that H4R3me2s expression was reduced following etravirine treatment (Figure 5k and Supplementary Figure 5c).

### Etravirine represses tumour growth in a 22Rv1 xenograft mouse model

**Figure 6.**
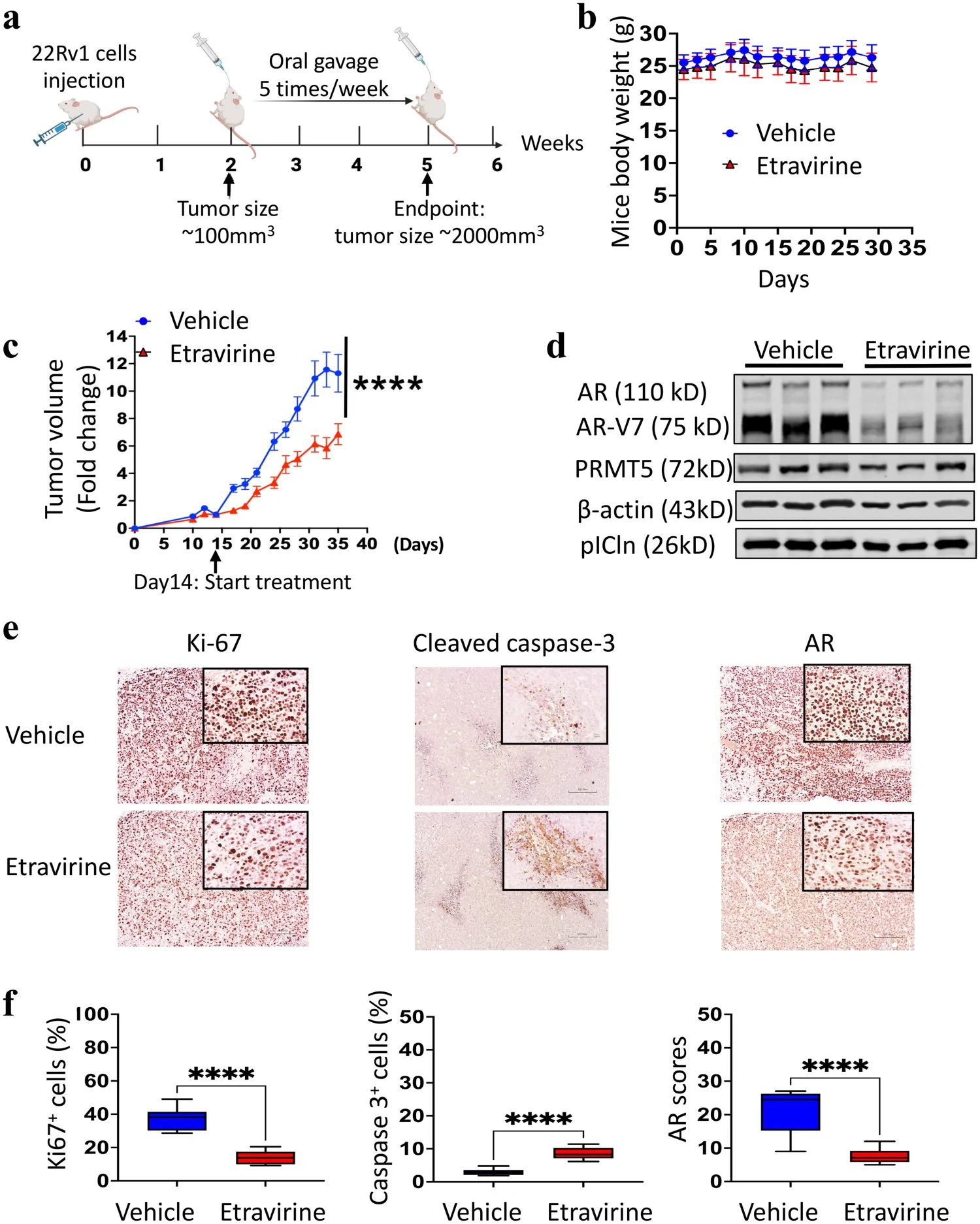
Etravirine impairs tumour growth in a 22Rv1 xenograft study**. a,** Workflow of 22Rv1 xenograft mice study. 22Rv1 cells were injected subcutaneously into the right flanks of surgically castrated male NRG mice (*n* = 10 mice/group). Vehicle or etravirine was orally administered with a dose of 100 mg/kg, 5 times/week. Mice were euthanized when tumours reached approximately 2000mm^3^. **b,** Body weight of NRG mice treated with either vehicle or etravirine. **c**, Volume fold change of 22Rv1 xenograft tumours treated with vehicle or with etravirine. Tumour volume is plotted as the means ± SEM. One-way ANOVA was performed to determine statistical significance. **d,** Representative Western blots of 22Rv1 tumour lysates with or without etravirine treatment. Each lane represents one mouse. **e**, Representative 4x (scale bar, 200 μm) and 40× (inset, scale bar, 20 μm) image of IHC probed with Ki-67, cleaved caspase-3, and AR from 22Rv1 xenograft tumour tissues from vehicle- and etravirine-treated mice. **f,** Quantification results from (**e**) using ImageJ software for Ki-67 and cleaved caspase-3, and H-score for AR. The t-test was performed to determine statistical significance. ns, nonsignificant, *P* > 0.05; *, *P* < 0.05; **, *P* < 0.01; ***, *P* < 0.001. ****, *P* < 0.0001.

To assess the *in vivo* applicability of our *in vitro* findings, we subcutaneously implanted 22Rv1 cells into male non-obese diabetic-Rag1(null)-γ chain(null) (NRG) immunocompromised mice after castrating them for two weeks. Given that etravirine is an FDA-approved drug with well-documented toxicity profiles^17^, we determined the dose and treatment period based on previous studies. Vehicle or etravirine was orally administered to randomly assigned mice bearing 22RV1 tumours (∼100 mm^3^). Throughout the treatment period, etravirine did not affect the body weight of the mice, compared to the vehicle group, indicating good tolerability (Figure 6b). By the time tumours in the vehicle group reached ∼2000 mm^3^ (approximately five weeks post-injection), mice in the etravirine group exhibited significant anti-tumour efficacy, as evidenced by reduced tumour growth (Figure 6c). Western blot analysis of tumour tissues suggested that AR and AR-V7 expression was downregulated in etravirine-treated tumour cells (Figure 6d). Additionally, immunohistochemistry (IHC) results demonstrated decreased expression of both AR and Ki-67 (proliferation marker) in the tumours of etravirine-treated mice. Ki-67 staining analysis revealed that tumours from etravirine-treated mice exhibited a significantly lower proliferative index compared to those from vehicle-treated mice. Cleaved caspase-3, an apoptosis marker, increased (Figure 6e). Cleaved caspase-3 staining indicated a marked induction of apoptosis in etravirine-treated mice. Collectively, these data have shown etravirine exhibits anti-proliferation activity and reduces AR and AR-V7 expression *in vitro* and *in vivo*.

## Discussion

PRMT5 has emerged as a promising therapeutic target for cancers in both preclinical studies and clinical trials^5^. Currently, most PRMT5 inhibitors focus on the enzyme’s catalytic or substrate-binding sites. However, because PRMT5 plays multiple critical functions and is tightly regulated in normal cells, inhibiting these active sites may lead to excessively broad inhibition of PRMT5 functions, significant intolerance, and toxicity in cancer patients. With ongoing clinical trials and limited efficacy data, the clinical benefits of these PRMT5 inhibitors remain uncertain. Unlike other PRMT family proteins, PRMT5 uniquely relies on cofactors, such as MEP50, pICln, RioK1, and COPR5. Targeting PRMT5-cofactor interactions presents an alternative approach that may expand the therapeutic window and reduce potential side effects linked to catalytic inhibition of PRMT5.

Inhibitors of PRMT5/PBM and PRMT5-adaptor interactions have recently been reported^11^^,^^12^. However, developing small molecules to disrupt PPI interfaces is challenging due to various factors influencing their efficacy. PPI interfaces often lack deep binding pockets, complicating molecular design. For instance, Compound 27 (BRD0639) stabilises its interaction with PRMT5 by forming a covalent bond with Cys278 in the groove at the PRMT5-PBM interface^11^. This covalent bond is essential for specificity but poses challenges *in vivo* due to potential off-target effects. Moreover, since the PBM is conserved among multiple cofactors, including pICln, COPR5, and RioK1, compounds targeting the PRMT5-PBM interaction may disrupt PRMT5′s binding with all three cofactors, potentially leading to high toxicity by excessively broad inhibition of PRMT5′s cellular functions.

Therefore, we sought to develop PRMT5 inhibitors that target the PPI sites unique to pICln, as we recently demonstrated that PRMT5/pICln interaction plays a critical role in the epigenetic reactivation of AR in prostate cancer^16^. To elucidate the interaction mode between PRMT5 and pICln, we used cryo-EM to determine the structure of the PRMT5/MEP50/pICln ternary complex using full-length proteins, from which a previously uncharacterised PRMT5 binding site, P4I, unique to pICln, was discovered. Notably, the P4I site contains a deep pocket that is essential for the PRMT5/pICln interaction, thereby providing a compelling structural basis for the development of non-covalent inhibitors that selectively disrupt this interface. Guided by these structural insights, we performed structure-based virtual screening targeting the P4I pocket on the PRMT5 N-terminal TIM-barrel domain from the (PRMT5)_4_(MEP50)_4_(pICln)_4_ complex at 3.03 Å resolution, which led to the identification of etravirine, an FDA-approved non-nucleoside reverse transcriptase inhibitor, as a non-covalent modulator that targets the PRMT5/pICln interface.

Our biochemical, biophysical, and structural assays confirmed that etravirine is a potent, specific inhibitor of the PRMT5/pICln interaction. We verified that etravirine interacts with PRMT5 and specifically disrupts PRMT5/pICln interaction, but not PRMT5/COPR5 interaction. We further determined the cryo-EM structure of the PRMT5/MEP50 complexed with etravirine, confirming that etravirine binds specifically at the PRMT5 P4I interface with a deep pocket region, and disrupts pICln interaction by competitive binding. Interestingly, the PRMT5/MEP50/etravirine structure and one class of PRMT5/MEP50/pICln structures both reveal a unique (PRMT5)_4_(MEP50)_3_ stoichiometry, a configuration that has never been reported to our best knowledge. Although only subtle conformational changes were observed in PRMT5/MEP50 with etravirine or pICln bound at the PRMT5 P4I site, the small conformational differences might be sufficient to favour the (PRMT5)_4_(MEP50)_3_ stoichiometry through a mechanism to be fully elucidated in future works.

We further verified etravirine’s efficacy with *in vitro* cellular assays and an *in vivo* xenograft mouse model of prostate cancer. In prostate cancer, abnormal reactivation of AR expression is a primary driver of CRPC progression and proliferation, underscoring the need for new therapeutic approaches to inhibit AR function. Our cellular assays indicated that etravirine suppresses AR and AR-V7 expression in a PRMT5/pICln PPI-dependent manner, effectively inhibiting prostate cancer cell proliferation. In line with our *in vitro* findings, etravirine reduced AR, and AR-V7 expression as well as significantly shrank tumours in xenograft mouse models. Together, these findings establish a proof-of-concept for a functional link between P4I targeting, disruption of the PRMT5/pICln interaction, suppression of AR and AR-V7 expression, and inhibition of prostate cancer progression in a xenograft model.

In addition to pICln, the PRMT5 adaptor proteins RioK1 and COPR5 engage PRMT5 through a distinct adaptor-binding site via their PRMT5-binding motifs (PBMs)^20^^,^^30^^,^^31^. This conserved PBM interface has been shown to facilitate substrate recruitment and enhances PRMT5-mediated methylation^31^. P4I-targeting compounds such as etravirine could be designed to selectively disrupt the PRMT5/pICln interaction without interfering with the interactions between PRMT5 and other adaptor proteins, including COPR5 and RioK1. This selective mechanism presents both advantages and limitations. On the one hand, the specificity of P4I targeting may minimise off-target effects associated with broader inhibition of PRMT5 adaptor interactions. On the other hand, because the canonical PBM-mediated interaction between PRMT5 and pICln remains intact, P4I-targeting compounds only partially impair, rather than completely abolish, the PRMT5/pICln interaction. Consistent with this mechanism, etravirine is likely to reduce AR expression by disrupting the dynamic occupancy of the PRMT5/pICln complex at AR regulatory regions rather than by inducing complete dissociation of the complex.

Given these characteristics, P4I-targeting compounds also provide a rationale for combination with existing therapies for prostate cancer. CRPC is a highly heterogeneous disease encompassing different molecular subtypes. AR signalling plasticity can drive CRPC and contribute to therapeutic resistance through multiple mechanisms including AR amplification, mutations, constitutively active splice variants, as well as AR‑independent phenotypes arising through neuroendocrine transdifferentiation^32^^,^^33^. Importantly, AR‑V7 is a well‑established mediator of resistance to next‑generation androgen receptor pathway inhibitors (ARIs) such as enzalutamide and abiraterone, because its constitutively active splice variant lacks the ligand‑binding domain targeted by these agents^34^. In this context, because etravirine reduces both full-length AR and AR-V7 through PRMT5/pICln modulation, P4I targeting may provide a complementary mechanism to conventional AR blockade. Combining etravirine with enzalutamide or abiraterone may therefore potentially enhance its efficacy in suppressing AR signalling. Further studies will evaluate etravirine in combination with ARIs using AR-V7-positive models, including long-term hormone-deprived culture systems and patient-derived xenografts, to evaluate potential synergy effect. P4I targeting may also have therapeutic implications beyond AR regulation. We previously demonstrated that PRMT5 and pICln cooperate to epigenetically activate genes involved in DNA double-strand break (DSB) repair across multiple cancer types. Reduced expression of either PRMT5 or pICln sensitises cancer cells to ionising radiation^24^. Therefore, combining etravirine with radiotherapy may provide synergistic antitumor effects in prostate cancer and other malignancies by simultaneously impairing DSB repair and enhancing treatment sensitivity. Additionally, we previously reported that targeting PRMT5 suppresses radiation-induced neuroendocrine differentiation and sensitises prostate cancer cells to radiation^35^. Expanding such evaluations to other AR‑independent models, such as NEPC, may be meaningful for rigorously defining the therapeutic scope of Etravirine. Thus, combination treatment with etravirine and radiotherapy represents another potentially valuable therapeutic strategy that needs further investigation.

The broader applicability of P4I targeting is supported by identification of additional compounds directed against this interface. In a separate study, we employed a machine learning–based virtual screening strategy to identify an additional P4I-targeting compound, Z319334062, which was shown to reduce symmetric dimethylarginine (SDMA) levels and inhibit cell viability in patient-derived glioblastoma cell lines^36^. Together with the present findings, these results suggest that targeting the P4I interface may represent a broadly applicable therapeutic strategy across multiple cancer types. Nevertheless, etravirine exhibits relatively modest binding affinity for PRMT5, and the current cryo-EM reconstruction does not provide sufficient resolution to define a high-confidence ligand-binding pose. Accordingly, etravirine should currently be regarded as a proof-of-concept P4I-targeting compound. Future efforts will focus on structure-guided medicinal chemistry optimisation of etravirine and related P4I-targeting compounds to improve their binding affinity, potency, and selectivity. Comprehensive pharmacokinetic and pharmacodynamic studies, together with further evaluation of efficacy and safety, will be required to establish the therapeutic potential of these compounds before clinical translation.

Drug repurposing is an attractive approach that extends the original medical application of approved drugs to new therapeutic uses. This strategy has proven effective and economical in accelerating drug discovery and development. Compared with newly discovered drugs, which require extensive clinical testing to ensure safety, drug repurposing offers several advantages, including lower risk, shorter timelines, and reduced resource requirements. Notable examples include aspirin, repurposed for antiplatelet therapy and colorectal cancer, and sildenafil, originally developed for hypertension but later rebranded as Viagra by Pfizer to treat erectile dysfunction^37^. In this study, we report an initial success in repurposing an approved HIV drug as a potential treatment for prostate cancer. We first used structure-based and computational modeling-guided drug discovery methods to screen FDA-approved drugs for molecules targeting the deep pocket region of the PRMT5 P4I interface, which led to the selection of etravirine, initially developed/approved to treat HIV infection^38^. Further biophysical, biochemical, structural, cellular, and animal assays have comprehensively verified the on-target binding and efficacy in inhibiting prostate cancer cell proliferation.

In summary, this study reports a cryo-EM structure-based drug discovery effort that has resulted in the development of a first-in-class inhibitor molecule, etravirine, for the treatment of prostate cancer by targeting the unique PRMT5/pICln PPI interface, P4I, newly discovered in this work. It also opens new possibilities for optimising etravirine and developing additional small molecules to target the P4I interface.

## Methods

### Protein expression and purification

The coding sequences for His-tagged PRMT5 and GST-tagged MEP50 were cloned into the pFastBac Dual vector. A PreScission protease cleavage site was added between the GST tag and MEP50. DH10Bac competent cells was used to produce bacmid. Baculovirus generation was carried out in Sf9 cells. The cells were harvested 60 h after baculovirus infection, followed by centrifugation, and stored at −80 °C until further use. Cell pellets were sonicated in lysis buffer (50 mM Tris pH 7.5, 150 mM NaCl, 10% glycerol, 1 mM EDTA, 1 mM PMSF, 0.1% X-100, 5 mM DTT) with the addition of 5 µl/ml DNaseI. The lysate was cleared by ultracentrifugation for 60 min at 48,000 g and the supernatant was incubated with an appropriate amount of equilibrated glutathione agarose (1 ml agarose per 20 ml supernatant) on an end-over-end rotator at 4 °C for 2h. Then the agarose was washed with 10 volumes of wash buffer 1 (10 mM Hepes pH 7.5, 150 mM NaCl, 5% glycerol). After the complex was eluted from glutathione agarose with elution buffer (10 mM Hepes pH 7.5, 150 mM NaCl, 5% glycerol, 10 mM L-Glutathione reduced), PreScission protease was added to remove the GST tag from MEP50 at 4 °C for 4h. Then the complex was incubated with Ni-NTA in incubation buffer (10 mM Hepes pH 7.5, 150 mM NaCl, 5% glycerol, 10 mM imidazole) on an end-over-end rotator at 4 °C for 1h. After Ni-NTA was washed with wash buffer 2 (10 mM Hepes pH 7.5, 150 mM NaCl, 5% glycerol, 10 mM imidazole), the complex was eluted with Ni-NTA elution buffer (10 mM Hepes pH 7.5, 150 mM NaCl, 5% glycerol, 250 mM imidazole). Protein complex from the Ni-NTA affinity column was then concentrated, purified with a Superdex 200 10/300 GL size-exclusion column pre-equilibrated with 10 mM Hepes, 150 mM NaCl, pH 7.5, 5% glycerol.

GST-tagged pICln was cloned into pETduet-1 vector. A PreScission protease cleavage site was added between the GST tag and pICln. The pICln-containing plasmid was transformed into E. coli strain BL21(DE3). pICln expression was induced with 0.2 mM IPTG for 16 h at 16 °C. The cells were collected by centrifugation and sonicated in lysis buffer containing 20 mM Tris-HCl, pH 8.0, 150 mM NaCl. Lysates were incubated with glutathione agarose. After washing with the buffer containing 20 mM Tris-HCl, pH 8.0, 150 mM NaCl, 1 mM DTT, the protein was cleaved by PreScission protease on column at 4 °C overnight. Then pICln was washed out from glutathione agarose with 10 mM Hepes, 150 mM NaCl, pH 7.5.

### Cryo-EM grid preparation and data collection

For PRMT5/MEP50/pICln, PRMT5/MEP50 at 1 mg/ml was mixed with pICln in a 1:4 molar ratio. 3 µL sample was applied to home-made graphene oxide-coated cryo-EM grids after glow discharging for 10 s. The grid was subsequently blotted for 15 s at 4 °C, 100% relative humidity, and plunge-frozen into liquid ethane using Vitrobot Mark IV. For PRMT5/MEP50/etravirine, PRMT5/MEP50 at 2 mg/ml was incubated with 100 µM etravirine (TMC125, MedChemExpress) and 100 µM PRMT5 inhibitor 16–19 F in 10 mM Hepes, 150 mM NaCl, pH 7.5, 5% DMSO for 16h and diluted with 10 mM Hepes, 150 mM NaCl, pH 7.5 to 1 mg/ml. 3 µL sample was applied to glow-discharged Quantifoil R1.2/1.3, 400 mesh grids and frozen with the same procedure as that for PRMT5/MEP50/pICln.

Both datasets were collected using a Titan Krios transmission electron microscope equipped with a post-column Gatan Quantum GIF energy filter and a Gatan K3 direct detector (Table S1). The slit width of the energy filter was set to be 20 eV. Movie stacks of PRMT5/MEP50/pICln were recorded in super-resolution mode with a pixel size of 0.4191 Å per super-resolution pixel at the specimen level, whereas movie stacks of PRMT5/MEP50/etravirine were recorded in super-resolution mode with a pixel size of 0.411 Å per super-resolution pixel at the specimen level. A total dose of 58.755 e/Å^2^ was fractionated into 51 frames for PRMT5/MEP50/pICln, and 95.015 e/Å^2^ was fractionated into 40 frames for PRMT5/MEP50/etravirine. The defocus range of both datasets was set between −0.7 µm and −2 µm.

### Cryo-EM image processing

Image processing was performed with the CryoSPARC v4.5 software^39^. The procedure for PRMT5/MEP50/pICln and PRMT5/MEP50/etravirine data processing was essentially the same. The super-resolution movies were Fourier-binned by a factor of 2, gain-normalised, dose-weighted, and aligned for beam-induced motion correction using Patch Motion Correction. Parameters of the contrast transfer function (CTF) were estimated using Patch CTF Estimation. The particles were manually picked with a subset of micrographs, then the templates generated by 2D classification were used to pick particles with Template Picker. The contaminant picks were removed by Inspect Picks. The particles were extracted with a box size of 400 pixels, but Fourier cropped to box size of 100 pixels. Two rounds of 2D classification were performed to clean up the particles. Then, the particles assigned to 2D classes with clear features were selected to reconstruct 3D structures. The initial models were built using Ab-Initio Reconstruction. Several rounds of Heterogeneous Refinement were performed to classify the particles. The classes with well-resolved 3D features were selected for Homogeneous Refinement. To increase map visual details, all the maps were sharpened with visually adjusted B factors.

To perform focused reconstruction of PRMT5/etravirine in the PRMT5/MEP50/etravirine structure, the particle datasets were symmetry-expanded using D2 symmetry. PRMT5 dimer in PRMT5/MEP50/etravirine structure was used to generate masks around the etravirine densities. The focused reconstruction had a nominal resolution of 2.93 Å with slightly clearer densities of etravirine.

### Model building and refinement

The crystal structure of PRMT5/MEP50 tetramer (PDB ID: 4GQB) was used as a starting model to rigid-body fit into the density maps using ChimeraX^40^. Then the models of PRMT5/MEP50 were manually trimmed, extended, and adjusted in Coot^41^. The model of pICln at the previously reported shallow PBM groove (PDB ID: 6V0O) was rigid body fit into the density and then manually adjusted in Coot, whereas the model of pICln at our newly identified binding site P4I was built *de novo* with Coot. Finally, both models of PRMT5/MEP50/pICln and PRMT5/MEP50/etravirine were refined in Phenix using real-space refinement with appropriate restraints^42^.

### Cell lines and cell culture

LNCaP, 22Rv1, COS1 cells were purchased from ATCC. COS1 cells were cultured in DMEM medium (Corning); LNCaP and 22Rv1 cells were maintained in RPMI medium (Corning). All culture media were supplemented with 10% foetal bovine serum (FBS, Atlanta Biologicals), 1% Penicillin/Streptomycin (Gibco), 1% sodium pyruvate (Corning), and 1% L-glutamine (Corning). Cells were routinely tested for mycoplasma contamination using the PCR Mycoplasma Detection Kit (Sigma). After thawing, cell lines were cultured for three passages before being used in experiments, and were maintained for no more than 30 passages. All cells were incubated at 37 °C and 5% CO_2_.

### Bimolecular fluorescence complementation (BiFC) assay

Stable LNCaP-6TR#2–1 cells were seeded at 50,000 cells/well into a 12-well plate and incubated for 24 h. Cells were transfected with 250 ng/well pcDNA-4TO-VC155-PICLN-PRMT5-NT292-VN BiFC plasmid (i.e. YFP signal) and 100 ng/well pFlag-cerulean (i.e. CFP signal) as a transfection control. After 24 h, DMSO or compound at the indicated concentrations was added to the respective wells. Doxycycline (1 μg/mL final concentration) was then added to each well. Cells were imaged using a Nikon TE-2000U microscope after 18 h of treatment. CFP and YFP images were acquired with MetaMorph software (Nikon) using a 20x objective. ImageJ software was used for analysis. Regions of interest (ROI) were selected in each cell, and the mean intensity was measured. The YFP/CFP ratio was calculated for etravirine and DMSO-treated cells. Then YFP/CFP ratio in etravirine treated cells was normalised to the YFP/CFP ratio in DMSO-treated cells to generate the BiFC efficiency. BiFC efficiency (YFP/CFP) was analysed essentially as described previously^16^^,^^25^.

### Proximity ligation assay (PLA)

LNCaP cells (0.1 million) were seeded on coverslips and treated with DMSO or varying concentrations of etravirine for 24 h. Cells were fixed with 4% paraformaldehyde for 10 min, permeabilized with 0.5% Triton X-100 in PBS for 10 min, and blocked with 1% bovine serum albumin and 5% goat serum for 1 h. Cells were then incubated with PRMT5 (mouse, Invitrogen MA5-32847) and pICln (rabbit, Abcam, ab192907) antibodies at 4 °C overnight in a humid chamber sealed with parafilm. The next day, two PLA probes (Duolink^®^ In Situ Red Starter Kit Mouse/Rabbit (DUO92101)) were diluted and incubated for 20 min at room temperature. The manufacturer’s instructions were followed for PLA ligase incubation, amplification, washing, and mounting with ProLong^®^ Antifade Kit (Invitrogen, P36980), followed by sealing with clear nail polish. Negative controls consisted of LNCaP cells treated only with PLA probes (no primary antibodies). Imaging was performed using a Nikon TE2000 inverted fluorescence microscope with a 40× objective.

### Co-Immunoprecipitation (Co-IP)

22Rv1 cells were seeded in 10 cm cell culture dishes and incubated until ∼80% confluence. Cells were then incubated with DMSO or etravirine at 5 μM, 10 μM, or 20 μM for 10 h. Cells were collected and lysed in Co-IP buffer (50 mM Tris-HCl, pH 7.4, 100 mM NaCl, 10 mM EDTA, 0.1% Triton X-100, 1 mM DTT, 1 mM PMSF, and protein inhibitors cocktail with 5 μg each of chymostatin, leupeptin, pepstatin A, antipan) on ice for 30 min. Cells were lysed by sonication (Branson Sonifier250set, Wilmington, NC, USA) with three 5-s cycles on ice. Lysates were centrifuged at 12,000 rpm for 15 min at 4 °C, and supernatants were collected. Total lysate (2 mg/mL) was incubated overnight at 4 °C with 4 μg of the proteins of interest, or a non-specific rabbit IgG control (Millipore Sigma N01-100UG) in the presence of DMSO or etravirine (5 μM, 10 μM, or 20 μM). Antibody-bound proteins were precipitated using Pierce Protein A agarose beads (Thermo Scientific 20333) for another 2 h at 4 °C. Supernatant was discarded, and 50 μL 2X SDS buffer was added to each sample. Samples were boiled at 95 °C for 5 min, and stored at −80 °C before Western blot analysis.

### Western blot

Cells lysates were prepared using Co-IP buffer or RIPA buffer (10 mM Tris-HCl pH 8.0, 5 mM EDTA, 1% Triton X-100, 0.1% Sodium Deoxycholate, 0.1% SDS, 140 mM NaCl, 5 μg/mL of each Chymostatin, Leupeptin, Pepstatin A, and antipan in DMSO, and 1 mM PMSF). Total protein concentration was measured using Pierce^™^ BCA Protein Assay Kit (Thermo Fisher, #23225). Approximately 20 μg of total protein was run on a 10–15% SDS-PAGE gel and transferred to a nitrocellulose membrane. Membranes were blocked with 5% milk and incubated with anti-AR rabbit antibody (Abcam, ab133273, 1:1000), anti-β-actin mouse antibody (Sigma, A1978 1:5000), anti-PRMT5 rabbit antibody (Millipore 07–405, 1:1000) or anti-CLNS1A rabbit antibody (Abcam, ab192907, 1:1000), anti-MEP50 mouse antibody (Invitrogen MA5-32970, 1:1000), anti-COPR5 rabbit antibody (Novus Biological, NBP2-30884, 1:1000), anti-Histone H4 mouse antibody (Abcam, ab31830, 1:1000) or anti-H4R3me2s rabbit antibody (Abcam, ab5823, 1:1000). Secondary antibodies (IRDye 800CW goat anti-rabbit IgG or IRDye^®^ 680RD goat anti-mouse IgG, LI-COR) were used for detection. Membranes were imaged using an LI-COR Odyssey CLx imager and analysed with ImageStudioLite software (LI-COR). The blue prestained protein standard with broad range (11–250 kDa) is from NEB (P7718).

### MTT assay

LNCaP, 22Rv1, and COS1 cells were seeded in 48-well cell plates (15,000 cells/well) and incubated for 24 h. Cells were treated with DMSO or etravirine at concentrations ranging from 40 μM to 0.625 μM, fed with fresh medium plus DMSO or drug every 48 h. Cells were subjected to MTT assay at the indicated time points. For MTT measurement, media were aspirated, and 70 μL of RPMI medium containing 0.5 mg/mL of fresh MTT (Sigma) was added to each well. After 4 h of incubation, 180 μL of DMSO was added to dissolve formazan. Absorbance was measured at 595 nm wavelength using a BioTek Synergy 4 plate spectrophotometer. Three technical replicates were included for each treatment group.

### RT-qPCR assay

LNCaP or 22Rv1 cells were seeded in 6 cm dishes and treated with either etravirine at different concentrations (2.5 μM, 5 μM, or 10 μM) or with DMSO for 24 h. Cells were then collected and lysed with 1 ml Trizol reagent (Ambion). RNA isolation was performed following the Trizol protocol. RNA purity was assessed, and integrity was verified via agarose gel electrophoresis. A total of 1 µg RNA was used to synthesise cDNA using Promega High-Capacity cDNA Reverse Transcription Kit (Promega, #4374966), following the manufacturer’s instructions. RT-qPCR was performed with FastStart Universal SYBR Green Master Mix (Thermo Fisher Scientific, #4385612) and detected on a QuantStudio 6 Flex (Thermo Fisher Scientific). Three technical replicates were included for all samples, and primer specificity was validated via melt curve analysis and agarose gel electrophoresis of the RT-qPCR product. Data analysis was conducted using QuantStudio design and analysis software (Thermo Fisher Scientific). All primers were used as previously described^16^.

### Xenograft tumour study

5 x 10^5^ 22Rv1 cells in 100 μl of PBS mixed with 100 μl Matrigel were implanted subcutaneously in 6 ∼ 8-week-old male non-obese diabetic-Rag1(null)-γ chain(null) (NRG) immunocompromised mice (10 mice/group) after castration for two weeks. Once tumour volumes reached ∼100 mm^3^, mice were randomised into two groups of approximately equal size: vehicle and etravirine treatment groups. Mice received oral administration of either 100 mg/kg etravirine or vehicle (10% DMSO, 44% PEG400, 1% Tween 80, and 45% saline solution) once daily, 5 times/week. Tumour volume was measured using ½ × L × W × H three times per week^15^. When control tumours reached ∼2000 mm^3^, tumours were resected for IHC, Western, and qPCR analysis. A Kaplan-Meier plot was generated to identify any survival advantage from etravirine treatment. Animal experiments were performed in the Biological Evaluation Facility of the Purdue University Institute for Cancer Research, approved by the Purdue University Animal Care and Use Committee (IACUC ID: 1112000342TR016).

### Immunohistochemistry (IHC)

Xenograft tumours were harvested upon termination of the mice due to tumour burden, and subjected to IHC staining. Tumours were fixed in 10% formalin for 24 h and paraffin-embedded. Tissue sections, 4 μm thick, were prepared using a Thermo microtome, mounted on charged slides, and dried in a 60 °C oven (Purdue University Histology Research Laboratory). Slides were deparaffinized after 2-h incubation at 65 °C, followed by three changes of xylene and rehydration through graded ethanol to water as previously described^15^. Antigen retrieval was conducted in a pressure cooker at 100 °C for 15 min using 10 mM Tris-HCl (pH 10). Slides were incubated with 3% hydrogen peroxide for 10 min, followed by blocking with 2.5% goat serum for 1 h. Then, primary antibody incubation was performed overnight at 4 °C with Ki-67 (Novus, NBP2-22112), cleaved caspase-3 (Cell Signalling, #9661) or AR (Abcam, ab133273) antibodies, each diluted 1:100 in TBST. After three TBST washes, slides were incubated with either goat anti-mouse (Jackson Immuno Research, #115–065-003) or goat-anti-rabbit secondary antibody (Jackson Immuno Research, #111–065-003) for 1 h. After washing with TBST three times, slides were treated with Vectastain elite ABC reagent (Vector, #PK-6100) for 30 min and developed using DAB substrate kit peroxidase (Vector, #SK-4100) for 5 min. Slides were then rinsed in water and counterstained with haematoxylin, dehydrated, and cleared before being sealed with mounting media. Images of IHC slides were captured using a Nikon microscope. Ki-67 and Cleaved Caspase 3 staining were analysed using Image J software. Semi-quantification of AR expression in the nucleus was performed by H-Score as described previously^43^.

### Justification for the use and number of animals

In vitro models are not sufficiently complex to approximate the complex biological functions of an intact vertebrate mammalian host. Therefore, in vivo models are required to study cancer biology in a context that includes tumour–host interactions and systemic effects. Mouse is a widely accepted and well-established model organism for such studies. Where previous applicable in vivo data are available, a power analysis is calculated using existing data to determine mice per group. In the absence of preliminary data on the efficacy of etravirine in inhibiting prostate cancer, we used a combination of published literature and statistical estimation to justify group sizes. Specifically, we consulted a published study of etravirine in an ovarian xenograft model, in which *n* = 5 mice per group was sufficient to detect significant differences in ovarian tumour growth^36^. To ensure adequate statistical power and rigour, we supplemented this with a formal power analysis using the “Sample size – Means” function from the sample size calculator (https://sample-size.net/). The parameters were set as follows: α (two-tailed) = 0.05, β = 0.2 (power = 80%), q1 = 0.5, q0 = 0.5, and S = 1. The effect size (E) was estimated as 1.5 based on reported differences in tumour size and weight from the referenced mouse study1. The calculator estimated a required sample size of 9 animals per group using a t-test, or 7 animals per group using a Z-test. To ensure sufficient power while accounting for biological variability and potential attrition (e.g. loss of animals during the study), we selected a group size of *n* = 10 mice. This number is consistent with commonly accepted practices in tumour biology studies and represents a balance between statistical rigour and ethical considerations to minimise animal use.

### Housing, feeding, and environmental enrichment details

Three on-site full-time veterinarians are available M-F 8–5 and 2 are on call 24/7 365 days/yr. Six vet techs are available on-site M-F during normal business hours for any animal care needs and all cages are checked daily for health and well-being of rodents. Up to 3 vet techs are available on weekends and holidays during normal hours and all cages are checked daily on weekends/holidays for health and well-being of rodents. Mice are housed in micro-isolator individually ventilated cages on organised racks with an auto watering system and provided feed pellets *ad libitum*. Shredded paper and housing huts are provided as enrichment to minimise fighting and distress.

### Information on analgesia, anaesthesia, and steps taken to minimise suffering

Our study design did not require the use of anaesthesia or analgesia for pain control. Animals were monitored at least twice weekly for humane euthanasia criteria. Euthanasia was performed by compressed CO_2_ inhalation followed by cervical dislocation per approved AVMA guidelines.

### A declaration of adherence to ARRIVE guidelines

We adhere to the ARRIVE guidelines to ensure studies are reported in enough detail to add to the knowledge base and that qualified individuals can repeat the employed methods. Notably, for randomisation in tumour studies, we use the minimisation strategy to account for the nuisance variable of irregular tumour size. This ensures all groups are arranged with tumours of roughly equivalent average volume and standard deviation. This permits variations in tumour outgrowth to be associated with treatment parameters and not differential initial starting tumour volumes^17^.

## Supplementary Material

Supplemental Material

## Data Availability

The data supporting the findings of this study are available within the article and its supplementary materials. In addition, the structural data are openly available in EMDB and PDB with the accession numbers for the cryo-EM maps and atomic coordinates as follows: PRMT5/MEP50 in complex with SAH and compounds 16–19 F and etravirine, EMD-70926/PDB 9OVY; PRMT5/MEP50:pICln complex at a 4:4:4 stoichiometric ratio, EMD-73799/PDB 9Z49; and PRMT5/MEP50:pICln complex at a 4:3:4 stoichiometric ratio, EMD-73801/PDB 9Z4A, respectively.
