# Supplementary material for: Cryo-EM structure-based discovery of etravirine as a specific inhibitor of PRMT5/pICln protein-protein interaction for prostate cancer treatment
Source: J Enzyme Inhib Med Chem. 2026 Sep 8;41(1):2727844. doi: 10.1080/14756366.2026.2727844 (PMC13556114; doi:10.1080/14756366.2026.2727844)
Supplement: Supplementary file 1 [file IENZ_A_2727844_SM3251.pdf]

	PRMT5:MEP50:pICln complex at a 4:4:4 stoichiometric ratio	PRMT5:MEP50:pICln complex at a 4:3:4 stoichiometric ratio	PRMT5:MEP50:Etravirine
**Data collection and processing**			
Magnification	105000		105000
Voltage (kV)	300		300
Electron exposure (e−/Å^2)	58.755		95.015
Defocus range (μm)	−0.6 to −2.0		−0.6 to −2.0
Pixel size at detector (Å/pixel)	0.838		0.822
Symmetry imposed	D2	C1	C1
Map resolution (Å)	3.03	3.59	3.12
FSC threshold	0.143		
**Refinement**			
Model composition			
Nonhydrogen atoms	29620	27332	27074
Protein residues	3780	3473	3368
Root mean square deviations			
Bond lengths (Å)	0.002	0.002	0.004
Bond angles (°)	0.477	0.483	0.933
**Validation**			
MolProbity score	1.49	1.51	1.28
Clashscore	3.45	3.66	4.21
Poor rotamers (%)	0	0	0
Ramachandran plot			
Favored (%)	94.88	94.83	97.63
Allowed (%)	5.12	5.17	2.28
Disallowed (%)	0	0	0

Information Classification: General
